# The Mediterranean Diet: Effects on Insulin Resistance and Secretion in Individuals with Overweight or Obesity

**DOI:** 10.3390/nu15214524

**Published:** 2023-10-25

**Authors:** Claudia Vetrani, Ludovica Verde, Annamaria Colao, Luigi Barrea, Giovanna Muscogiuri

**Affiliations:** 1 Dipartimento di Scienze Umanistiche, Università Telematica Pegaso, Centro Direzionale, Via Porzio, Isola F2, 80143 Naples, Italy; luigi.barrea@unina.it; 2 Centro Italiano Per La Cura E Il Benessere del Paziente Con Obesità (C.I.B.O), University of Naples “Federico II”, 80131 Naples, Italy; ludovica.verde@unina.it (L.V.); operafederico2@gmail.com (A.C.); giovanna.muscogiuri@unina.it (G.M.); 3 Department of Public Health, University of Naples “Federico II”, Via Sergio Pansini 5, 80131 Naples, Italy; 4 Dipartimento di Medicina Clinica e Chirurgia, Diabetologia ed Andrologia, Unità di Endocrinologia, Università Federico II, Via Sergio Pansini 5, 80131 Naples, Italy; 5 Cattedra Unesco “Educazione Alla Salute E Allo Sviluppo Sostenibile”, Università Federico II, 80131 Naples, Italy

**Keywords:** the Mediterranean diet, overweight, obesity, insulin resistance, insulin secretion, insulin sensitivity, HOMA, OGTT, β-cell function

## Abstract

High adherence to the Mediterranean Diet (MD) is associated with a lower risk of type 2 diabetes. However, it is less clear whether the different MD food items might influence specific biological functions related to glucose tolerance, i.e., insulin resistance (IR) and/or secretion (IS). Thus, this cross-sectional study aimed to investigate the relationship between adherence to MD and IR, insulin sensitivity, and IS in individuals with overweight/obesity. Participants (62 individuals; 7M/55F; mean age 49 ± 15 years; mean BMI 35.8 ± 6.7 kg/m²) underwent a 75 g oral glucose tolerance test (OGTT) to assess plasma glucose and insulin concentrations. These parameters were used for the calculation of validated IR indices (Homeostatic Model Assessment for Insulin Resistance (HOMA-IR), Homeostatic Model Assessment for β-cell function (HOMA-β)), as well as insulin sensitivity indices (insulin sensitivity index (ISI), oral glucose insulin sensitivity (OGIS)). MD adherence was gauged using the PREDIMED questionnaire. Bivariate correlations were used to highlight the association between OGTT-derived indices and MD adherence (PREDIMED score) or specific foodstuffs related to MD. Despite there being no significant differences in BMI, impaired fasting glucose (IFG), and impaired glucose tolerance (IGT), the high MD adherence group presented lower HOMA-IR (*p* = 0.022) and higher ISI (*p* = 0.033) compared to other groups. High MD adherence was inversely correlated with HOMA-IR (r = −0.400; *p* = 0.004) and directly correlated with ISI (r = 0.296, *p* = 0.039). Fish consumption, a key component of MD, exhibited significant associations: it was directly correlated to ISI (r = 0.394, *p* = 0.005) and inversely related to HOMA-IR (r = −0.327, *p* = 0.019) and β-cell function (r = −0.489, *p* < 0.001). In conclusion, a high MD adherence, and in particular the consumption of fish, is associated with a decreased IR in individuals with overweight/obesity.

## 1. Introduction

Overweight and obesity are recognized as chronic conditions that are associated with several comorbidities, namely type 2 diabetes (T2D), hypertension, dyslipidemia, cardiovascular diseases (mainly coronary heart disease and stroke), and some cancers [1,2,3]. These comorbidities reduce the quality of life and life span and increase public health costs [4].

Insulin resistance (IR) has been appointed as one of the main drivers of obesity-related complications [5]. It is defined as an impaired response to insulin stimulation of target tissues (primarily liver, muscle, and adipose tissue) that induces hyperinsulinemia [6]. Indeed, to compensate for IR, insulin secretion from pancreatic β-cells increases while hyperglycemia occurs. Over time, this chronic condition can lead to prediabetes, identified as impaired fasting glucose (IFG) or impaired 2 h post-challenge plasma glucose (i.e., impaired glucose tolerance, IGT) [7,8]. On the other hand, IR is a major cardiovascular risk factor in individuals without T2D, independently of other risk factors [9]. Notably, several studies reported that IR can be detected 10 to 20 years before the onset of T2D and cardiovascular diseases [10,11,12]. In addition, prospective cohort studies demonstrated that IR can also be detected in adults with overweight [1,13].

According to the latest reports, more than one-third of adults have prediabetes, with 374 million individuals (7.5%) presenting with IGT in 2019, and expected 8.0% (454 million) and 8.6% (548 million) by 2030 and 2045, respectively [14]. Nonetheless, most individuals are unaware they have the condition [13]. Therefore, the detection and treatment of IR or prediabetes are of paramount importance to prevent cardiometabolic complications [15].

IR and prediabetes are triggered by multiple factors such as excess body fat (mainly visceral deposition), unhealthy diets, low physical activity, and a family history of T2D [14,16].

The recommended first-line approach for the prevention of IR and prediabetes relies on lifestyle changes, including adequate physical activity and healthy eating habits [14,16]. Among the available nutritional patterns, adherence to the Mediterranean diet (MD) has been associated with a reduced risk of T2D [17,18,19]. In particular, a dose-response meta-analysis has detected a 14% lower T2D risk for each 2-point increment in adherence to the MD score [17]. These effects are triggered by the nutritional profile of MD that derives from the combination of specific foodstuffs intake during the week [20]. More in detail, MD is characterized by a consistent intake of plant-based foods (fruits, vegetables, wholegrain, legumes, and nuts), with extra-virgin olive oil (EVOO) as the main source of fat, moderate consumption of animal protein and fat (preferring fish, eggs, and low-fat dairies), and restricted intake of sweets and processed foods. Therefore, energy intake is provided by non-refined carbohydrates (55–60%), 30–35% from fat, and ~15% from protein [21]. Low-glycemic index foods (i.e., wholegrain-based products and legumes) are the main carbohydrate sources, while sugar intake is less than 10% by limiting sweets and sugar-sweetened beverages. As for fat quality, monounsaturated fatty acids (MUFA, ~19%) are higher than saturated fatty acids (SFA, ~9%) and polyunsaturated fatty acids (PUFA, ~5%), while cholesterol is 300 mg/day [22]. According to MD, plant protein intake should be higher than animal protein. Finally, significant amounts of vitamins, minerals, and other phytochemicals are provided by MD [20].

Nevertheless, it is poorly known whether the beneficial effect is triggered by the whole pattern or whether a specific item of MD has a specific role in the different biological functions, i.e., IR and/or insulin sensitivity and secretion. 

Against this background, the primary aim of this cross-sectional study was to evaluate the association between adherence to MD and indices of IR, insulin sensitivity, and secretion in individuals with overweight or obesity. The secondary aim was to assess whether the food cluster or a single MD food was associated with IR and/or insulin sensitivity and secretion.

## 2. Materials and Methods

### 2.1. Study Design, Setting, and Participants

This cross-sectional observational study was carried out according to the Strengthening the Reporting of Observational Studies in Epidemiology (STROBE) statement checklist [23].

The study was approved by the Local Ethical Committee (no. 05/14) and carried out in accordance with the Declaration of Helsinki for experiments involving humans. All the study participants provided written informed consent.

From January 2022 to January 2023, all patients attending the Obesity Centre C.I.B.O (Centro Italiano Per La Cura E Il Benessere del Paziente Con Obesità) of Federico II University Hospital (Naples, Italy) were screened for eligibility according to the following criteria: both genders, age 18–65 years, body mass index (BMI) > 27.0 kg/m^2^, and stable body weight (change < ±10% of body weight) in the 3 months before the study. We excluded participants presenting one or more of the following conditions: breastfeeding or pregnancy, type 1 diabetes, T2D, cardiovascular or cerebrovascular diseases (i.e., coronary heart disease, stroke, or revascularization), kidney and hepatic failure, chronic inflammatory diseases, and cancers. In addition, we excluded participants who are currently undergoing or have a history of treatment within 3 months before the study with the following medications: (a) glucose-lowering therapy (such as metformin, dipeptidyl peptidase 4 inhibitors, sodium–glucose cotransporter 2 inhibitors, pioglitazone); (b) glucagon-like peptide—1 receptor agonist, orlistat, naltrexone/bupropion; (c) glucocorticoids; (d) drugs that may cause a significant weight gain; and (e) antipsychotic medications. 

### 2.2. Lifestyle Habits and Anthropometric Measurements

All participants were asked for demographic information, personal medical history (i.e., diseases and medications), and lifestyle habits (smoking, physical activity, and alcohol use). As previously reported [24,25,26], current smokers included individuals smoking at least one cigarette/day, former smokers who had ceased smoking at least one year before the study, and non-current smokers as the remaining individuals. Physically active individuals included participants engaged in at least 30 min/day of any type of physical exercise (YES/NO). 

As for anthropometric measurements, body weight, height, and waist circumference (WC) were measured according to standardized procedures as reported in [27,28]. Weight and height were used for the assessment of BMI. Overweight and obesity were classified according to the World Health Organization criteria: overweight (25.0–29.9 kg/m^2^), obesity class I (30.0–34.9 kg/m^2^), obesity class II (35.0–39.9 kg/m^2^), and obesity class III (≥ 40.0 kg/m^2^).

### 2.3. Adherence to the Mediterranean Diet

Adherence to the MD was assessed by the PREDIMED questionnaire [29]. Briefly, it consists of 14 items related to the main MD characteristics (intake and amount of EVOO, frequency of fruit, vegetables, nuts, legumes, red meat, poultry, fish, animal fat, sweetened beverages, sweets, and sofrito). PREDIMED score was calculated by assigning a score of 1 and 0 for each item. The same face-to-face interview with a skilled nutritionist was used, as in previous studies [21,30]. According to the PREDIMED score, participants were classified as follows: score 0–5, lowest adherence; score 6–9, average adherence; and score ≥10, highest adherence [29].

### 2.4. Oral Glucose Tolerance Test

All participants underwent a 75 g OGTT with blood sampling with EDTA tubes at 0, 30, 60, 90, and 120 min. Plasma samples were stored at −80 °C until the analysis. Plasma glucose, insulin, and glycated hemoglobin (HbA1c) were assessed in the central biochemistry laboratory of the Federico II University Hospital. The Roche Modular Analytics System was used to perform all biochemical analyses. The Immunolite Diagnostic Products Co., Los Angeles, CA, USA with a solid phase chemiluminescent enzyme immunoassay kit was used to measure insulin concentrations. The intra- and inter-test coefficient of variation values are <7% for all tests performed.

### 2.5. Calculation of Derived Indices of Insulin Sensitivity and Secretion

Fasting indices were evaluated using the Homeostatic Model Assessment (HOMA-IR) as a marker of reduced insulin sensitivity ((fasting glucose, mg/dL × fasting insulin, mU/L)/405), whereas insulin secretion capacity was calculated as the HOMA for β cell function (HOMA-β: (20 fasting insulin, pmol/L)/(fasting glucose, mmol/L − 3.5)) [31]. For post-load evaluation, the total area under the curve (AUC) was calculated by the trapezoidal method. After glucose load, insulin action was evaluated by the 120 min oral glucose insulin sensitivity (OGIS) method [31], whereas the insulin sensitivity index (ISI) was calculated according to the method by Matsuda [32]. Insulin secretion was calculated as β cell function (insulin 0–120 AUC/glucose 0–120 AUC ratio) [31], whereas the insulinogenic index was calculated as a marker of first-phase insulin secretion, according to Pacini et al. [31]. The disposition index was calculated as the product of measures of insulin sensitivity and first-phase insulin secretion, as a measure of global glucose tolerance [33]. According to the American Diabetes Association, IFG was diagnosed with fasting plasma glucose ≥100 and <126 mg/dL while IGT was defined as 2 h plasma glucose ≥140 and <200 [7].

### 2.6. Sample Size Calculation and Statistical Analysis

The sample size was calculated to detect a 30% difference in the insulin sensitivity between the three groups of adherences to MD, with an 80% power at a 5% significance level. The estimated change is clinically based, corresponding to the differences observed between individuals with insulin resistance and insulin-sensitive individuals after a 2 h glucose tolerance test (OGTT with 75 g) [34]. In addition, considering a 40% non-response rate or missing information, we included 62 individuals in the study. Continuous variables were expressed as mean ± standard deviation (SD) whereas categorical variables were reported as numbers and percentages (%). Shapiro–Wilk test was used to test data distribution and variables not normally distributed were analysed after logarithmic transformation (Log10). Differences between groups were analysed by analysis of variance (one-way ANOVA) and *post hoc* analyses (least significant difference test, LSD). Chi-squared test for independence was used to assess the association between outcomes and categorical variables. Correlations between study variables were performed using Pearson’s correlation (continuous variables) or Spearman rank correlation (categorical variables). All analyses were adjusted for BMI as a potential confounder. Effect size was evaluated as Hedge g (0.2 small, 0.5 medium, and 0.8 large effect size) [35].

A *p* value < 0.05 was considered significant. Statistical analysis was performed according to standard methods using the Statistical Package for Social Sciences software 26.0 (SPSS/PC; SPSS, Chicago, IL, USA).

## 3. Results

The study population consisted of 62 participants (49 ± 15 years, mean BMI 35.8 ± 6.7 kg/m^2^): 7 men (11%), and 55 women (89%). The mean PREDIMED score was 7.60 ± 2.2. According to the PREDIMED score, 12 individuals (19.4%) belonged to the “low adherence” group, 35 (56.5%) to the “intermediate adherence” group, and 15 participants (24.2%) to the “high adherence” group.

The main demographic and clinical characteristics of the three groups are reported in Table 1. Participants in the “high adherence” group were significantly older than those belonging to the “low adherence” group (*p* = 0.026, LSD *post hoc* analysis for multiple comparisons). No differences were observed for sex distribution, BMI classes, disease prevalence, and lifestyle habits (physical activity, smoking, and alcohol use) among the three groups. As for glucose tolerance, the three groups were similar for HbA1c concentrations and the prevalence of IFG or IGT.

Fasting and post-load plasma glucose, insulin, and OGTT-derived indices of insulin sensitivity and secretion in the three groups are reported in Table 2. Fasting plasma glucose concentrations did not differ among the three groups, whereas fasting plasma insulin concentrations were significantly lower in the “high adherence” group than the other groups (*p* = 0.025 and *p* = 0.029, “low adherence” and “intermediate adherence”, respectively, LSD *post hoc* analysis for multiple comparisons). This translated into a significant difference in HOMA-IR that was lower in the “high adherence” group than the other groups (*p* = 0.012 and *p* = 0.019, “low adherence” and “intermediate adherence”, respectively, LSD *post hoc* analysis for multiple comparisons). As for post-load indices, insulin sensitivity (evaluated by ISI) was significantly higher in the “high adherence” group than the other groups (*p* = 0.016 and *p* = 0.037, “low adherence” and “intermediate adherence”, respectively, LSD *post hoc* analysis for multiple comparisons). An opposite trend was observed for the insulin secretion (evaluated by β-cell function), being β-cell function lower in the “high adherence” group than in the “intermediate adherence” group (*p* = 0.006, LSD *post hoc* analysis for multiple comparisons). No significant difference in the β-cell function between the “high adherence” and the “low adherence” groups was observed (*p* = 0.207, LSD *post hoc* analysis for multiple comparisons). These results remained significant, also adjusting for BMI (HOMA-IR *p* = 0.044; ISI *p* = 0.045; β-cell function *p* = 0.022).

Moreover, the analyses of the effect size confirmed the significant differences observed in fasting plasma insulin and indices of glucose tolerance (HOMA-IR, ISI, and β-cell function) among the MD groups being the Hedge’s g more than 0.5 in all comparisons (Table 3).

According to the bivariate correlation analyses, the PREDIMED score was directly associated with ISI (r = 0.296, *p* = 0.039), whereas an inverse correlation between the PREDIMED score and HOMA-IR was detected (r = −0.400; *p* = 0.004). As for the specific MD items, fish intake associated with HOMA-IR (r = −0.327, *p* = 0.019), ISI (r = 0.394, *p* = 0.005), and β-cell function (r = −0.489, *p* < 0.001). These associations remained significant also adjusting for BMI (ISI r = 0.348, *p* = 0.012) and β-cell function (r= −0.355, *p* = 0.024), except for HOMA-IR (r = −0.280, *p* = 0.047). All correlations presented a moderate magnitude of association among the variables.

## 4. Discussion

The present study showed that high adherence to MD was associated with a lower IR both at fasting and after glucose load (measured by HOMA-IR and ISI, respectively) in individuals with overweight or obesity. This effect was mainly associated with the consumption of fish.

In line with these results, a recent meta-analysis of 46 randomized clinical trials with omega-3 fatty acids (*n* = 4991 patients with T2D) showed an improvement in HbA1c concentrations [36]. Interestingly, in a meta-analysis evaluating the effect of fish oil supplementation (*n* = 820 patients with T2D), no difference in glycemic control was observed [37]. This could suggest that the source of omega-3 fatty acids might have a pivotal role in determining the effect on glucose tolerance.

Multiple mechanisms could explain the beneficial effect of omega-3 fatty acids [38,39,40]. Firstly, in vitro and in vivo studies demonstrated that omega-3 fatty acids can reduce lipolysis in the adipose tissue, thus reducing the efflux of free fatty acids (FFA) into circulation. The excess of FFA promotes the onset of IR by affecting insulin signaling [38]. Therefore, omega-3 fatty acids can counteract FFA thus limiting their effect on IR. On the other hand, omega-3 fatty acids have been shown to promote mitochondrial biogenesis and upregulate genes involved in fatty acid oxidation, while reducing lipogenesis [39]. Overall, these effects can contribute to reducing fat deposition in adipose tissue. Finally, omega-3 fatty acids can reduce low-grade inflammation by virtue of their anti-inflammatory properties. This effect has been associated with the increase in adiponectin secretion, the adipokine with the great insulin-sensitizing effect [40].

Another explanation could be related to the indirect reduction of SFA which has shown detrimental effects on insulin sensitivity. Indeed, in a randomized controlled trial in individuals without T2D, the substitution of SFA for PUFA induced a significant improvement in insulin sensitivity assessed by hyperinsulinaemic-euglycaemic clamps, the gold standard method for the evaluation of this parameter [41].

Another finding of the present study was that β-cell function was lower in the high adherence to MD group as compared to the other groups, but the difference reaches the conventional statistical difference only in the comparison with the intermediate adherence to MD group. This finding might be explained by the higher prevalence of obesity class II and III in the intermediate adherence to MD group (also related to a higher WC). Indeed, it is known that higher BMI values are related to a more pronounced IR, and it could lead to a more rapid detrimental effect on the β-cell function, likely due to the higher subclinical inflammation [42,43]. Unfortunately, we did not measure inflammatory markers in this study to test this hypothesis.

Previous studies with different experimental designs explored the effects of PUFA on glucose metabolism with unclear results [44,45,46,47]. Therefore, the inverse association between fish intake and β-cell function might be explained by the reduction of lipotoxicity due to the exposure to fatty acids, as shown by in vitro studies [44,45], but might also reflect a mechanism of preservation of β-cell function [44]. Therefore, further studies are needed to establish the pathophysiological consequence of the reduction of β-cell function.

The results of the present study might have relevant clinical implications since the current guidelines underline the pivotal role of lifestyle changes as preventive strategies to reduce prediabetes and IR-related complications. In this context, MD could be a reliable and feasible tool to achieve the aim. Another strength of this study is the analyses of fasting and post-load indices of glucose tolerance. Indeed, glucose tolerance is a complex condition that relies upon the interplay between insulin sensitivity and insulin secretion [31]. The gradual impairment of these two processes leads to glucose intolerance and type 2 diabetes but it is still under debate which abnormality precedes the other one. Consequently, the most recent studies recommended regular monitoring of fasting and post-load glucose tolerance in individuals with high metabolic risk [48,49,50]. Therefore, our studies might give a better understanding of the mechanisms linking MD and T2D risk. 

A limitation of the present study is the cross-sectional design that does not allow us to establish any cause–effect relationship between the evaluated parameters. Nevertheless, the methodology used in this study (validated OGTT-derived indices) provided new insights into the potential mechanisms of action behind the association between MD and glucose tolerance. Further studies are needed to establish what component of fish might drive the beneficial effect on IR.

In addition, no information on dietary composition was collected in the present study. However, the adjustment for BMI could also help to correct the results for energy intake as the main determinant of the energy balance since no difference in physical exercise was observed, thus reducing potential confounders. It is worth mentioning that the analyses of dietary composition would have allowed a multiple regression analysis to better investigate the role of dietary components on IR and IS.

## 5. Conclusions

A high MD adherence is associated with lower insulin resistance in individuals with overweight/obesity. The effect is mainly related to fish consumption but the mechanisms underlying this association must be investigated in clinical trials. Nonetheless, MD could be a reliable and feasible tool for the prevention of insulin resistance-related complications in individuals with overweight/obesity.

## Figures and Tables

**Table 1:** Main demographic and clinical parameters of study population according to adherence to MD.

Parameters	Low Adherence to MD *n* = 12	Intermediate Adherence to MD *n* = 35	High Adherence to MD *n* = 15	*p* Value *
Sex (M/F)	2/10	3/32	2/13	0.428
Age (years)	43 ± 14	48 ± 14	55 ± 14 ^a^	0.073
Physical activity (*n*,%)	1 (8)	4 (11)	4 (27)	0.298
Smoking (*n*, %)	1 (8)	6 (17)	1 (7)	0.709
Alcohol use (*n*,%)	0 (100)	9 (26)	5 (33)	0.196
BMI (kg/m^2^)	38 ± 9	36 ± 6	33 ± 7	0.173
Overweight (*n*, %)	0 (0)	0 (0)	1 (7)	0.076
Obesity I (*n*, %)	4 (33)	4 (11)	5 (33)	
Obesity II (*n*, %)	2 (17)	11 (31)	3 (20)	
Obesity III (*n*, %)	6 (50)	9 (26)	4 (27)	
WC (cm)	112 ± 20	114 ± 15	106 ± 14	0.229
Hypertension (*n*, %)	2 (17)	8 (23)	5 (33)	0.580
Dyslipidaemia (*n*, %)	1 (8)	8 (23)	6 (40)	0.155
Metabolic Syndrome (*n*, %)	1 (8)	5 (14)	4 (27)	0.395
IFG (*n*, %)	2 (17)	6 (17)	1 (7)	0.586
IGT (*n*, %)	0 (0)	5 (14)	0 (0)	0.136
HbA1c (%)	5.5 ± 0.4	5.6 ± 0.5	5.6 ± 0.5	0.869

BMI, body mass index; WC, waist circumference; IFG, impaired fasting glucose; IGT, impaired glucose tolerance; HbA1c, glycated haemoglobin. Data are expressed as mean ± standard deviation or *n* (%). * one-way ANOVA for continuous variables and χ^2^ test for categorical variables. ^a^ *p* < 0.05 vs. low adherence (at least-significant-difference *post hoc* analysis for multiple comparisons).

**Table 2:** Fasting and post-load plasma glucose, insulin, and OGTT-derived indices of insulin sensitivity and secretion according to adherence to MD.

Parameters	Low Adherence to MD *n* = 12	Intermediate Adherence to MD *n* = 35	High Adherence to MD *n* = 15	*p* Value *
Fasting plasma glucose (mg/dL)	95 ± 5	96 ± 12	92 ± 7	0.125
Fasting plasma insulin (μU/mL)	18 ± 6	16 ± 9	10 ± 4 ^a,b^	0.043
2 h glucose AUC (mg/dL·120 min)	227 ± 10	246 ± 12	238 ± 13	0.602
2 h insulin AUC (μU/mL·120 min)	131 ± 64	166 ± 94	102 ± 50	0.058
Fasting indices				
HOMA-IR	4.1 ± 1.2	3.8 ± 2.1	2.3 ± 0.9 ^a,b^	0.022
HOMA-β	1472 ± 676	1448 ± 1247	983 ± 605	0.402
Post-load indices				
OGIS (mL × min^−1^ × m^−2^)	395 ± 60	388 ± 72	440 ± 52	0.137
ISI ((mg/dL)^2^/(μU/mL)^2^)^−1/2^)	1.2 ± 0.2	1.5 ± 1.2	2.4 ± 1.5 ^a,b^	0.033
Insulinogenic index (μU/mg)	1.3 ± 0.3	1.5 ± 1.0	1.6 ± 1.5	0.891
β-cell function (μU/mg)	0.58 ± 0.3	0.73 ± 0.4	0.42 ± 0.2 ^b^	0.020
Disposition index	0.09 ± 0.03	0.10 ± 0.07	0.16 ± 0.11	0.204

Data are expressed as mean ± standard deviation or *n* (%). AUC, area under the curve; HOMA-IR, Homeostatic Model Assessment for Insulin Resistance; HOMA-β, Homeostatic Model Assessment for β-cell function; ISI, insulin sensitivity index; OGIS, oral glucose insulin sensitivity. * one-way ANOVA for continuous variables. ^a^ *p* < 0.05 vs. low adherence (at least-significant-difference *post hoc* analysis for multiple comparisons). ^b^ *p* < 0.05 vs. intermediate adherence (at least-significant-difference *post hoc* analysis for multiple comparisons).

**Table 3:** Effect size * of fasting plasma insulin, HOMA-IR, ISI, and β-cell function.

Parameters	High Adherence to MD vs. Low Adherence to MD		High Adherence to MD vs. Intermediate Adherence to MD	
	Mean Difference (95% CI)	Hedge’s *g*	Mean Difference (95% CI)	Hedge’s *g*
Fasting plasma insulin (μU/mL)	−7.90 (−14.5–−1.29)	1.61	−6.21 (−11.5–−0.88)	0.77
HOMA-IR	−1.88 (−3.35–−0.41)	1.63	−1.56 (−2.74–−0.37)	0.76
ISI ((mg/dL)^2^/(μU/mL)^2^)^−1/2^)	0.91 (−0.03–1.84)	1.06	0.57 (−0.08–−1.22)	0.79
β-cell function (μU/mg)	−0.16 (−0.44–0.11)	0.20	−0.24 (−0.43–−0.05)	0.87

* Hedge’s *g* effect size: 0.2 small, 0.5 medium, and 0.8 large.

## Data Availability

The datasets used and/or analyzed during the current study are available from the corresponding author upon reasonable request.
